# Serum Levels of TNF-***α***, IL-12/23 p40, and IL-17 in Psoriatic Patients with and without Nail Psoriasis: A Cross-Sectional Study

**DOI:** 10.1155/2014/508178

**Published:** 2014-12-31

**Authors:** Aikaterini Kyriakou, Aikaterini Patsatsi, Timoleon-Achilleas Vyzantiadis, Dimitrios Sotiriadis

**Affiliations:** ^1^2nd Department of Dermatology and Venereology, Papageorgiou General Hospital, Aristotle University School of Medicine, Ring Road Thessalonikis, Nea Efkarpia, 56403 Thessaloniki, Greece; ^2^1st Department of Microbiology, Aristotle University School of Medicine, 54124 Thessaloniki, Greece

## Abstract

Nail involvement has started playing a major role in the overall assessment and management of psoriatic disease. Biologics indicated for moderate to severe chronic plaque psoriasis are shown to be beneficial in nail disease. This study aimed to assess and compare the serum levels of TNF-*α*, IL-12/23 p40, and IL-17 in psoriatic patients with and without nail involvement. 52 consecutively selected patients with chronic plaque psoriasis were included in this cross-sectional study. Patients were studied and analyzed after they had been divided into 2 groups regarding the presence (*n* = 24) or not (*n* = 28) of nail psoriasis. The mean serum levels of TNF-*α* were significantly higher in the group of psoriatic patients with nail lesions compared to those without (*t*-test; 5.40 ± 1.17 versus 3.80 ± 1.63, *P* = 0.026). However, the median serum levels of both IL-12/23 p40 (Mann-Whitney; 92.52 (34.35–126.87) versus 150.68 (35.18–185.86), *P* = 0.297) and IL-17 (Mann-Whitney; 28.49 (0.00–28.49) versus 8.59 (0.00–8.59), *P* = 0.714) did not significantly differ between the 2 groups. These results confirm the important role of TNF-*α* in the pathogenesis of nail psoriasis and may suggest that anti-TNF agents could be more beneficial in psoriatic nail disease than agents targeting IL-12/23 p40 or IL-17 and its receptors.

## 1. Introduction

Psoriasis is a chronic inflammatory disease that affects the skin, joints, and nails [1]. Nail involvement is a common manifestation of psoriasis, since most psoriatic patients will develop nail lesions at some point of their lives [2, 3]. It often causes functional impairment and psychological handicap leading to alterations in the quality of life [4–6]. Therefore, nail evaluation has been established in the assessment and management of psoriatic patients.

While pathogenesis of psoriasis has become better understood, tumor necrosis factor-*α* (TNF-*α*), IL-12, IL-23, and IL-17 seem to play key roles in the development of the immune response seen in psoriasis [7]. In light of these discoveries, biological agents, which target these cytokines and their receptors, have enriched the therapeutic armamentarium for treating psoriasis. Focusing on nail psoriasis, data up to date are encouraging and biologics indicated for moderate to severe chronic plaque psoriasis are shown to be beneficial in nail disease [3, 7–13]. However, which class of biologics is the most efficacious in nail psoriasis, anti-TNF-*α* agents, ustekinumab binding the shared p40 subunit of IL-12 and IL-23 or upcoming agents targeting IL-17 and its receptor, is still under investigation.

The aim of this study was to assess and compare the serum levels of TNF-*α*, IL-12/23 p40, and IL-17 in psoriatic patients with and without nail involvement.

## 2. Materials and Methods

A cross-sectional, hospital-based study was performed in which 52 consecutively selected patients with chronic plaque psoriasis divided into 2 groups, according to whether or not they had nail disease, were compared. Subjects above the age of 18 with clinically diagnosed active, chronic plaque psoriasis and PASI < 12, who had not received any systematic antipsoriatic treatment or any topical treatment against cutaneous or nail psoriasis for at least a year, were eligible to participate in this study. Exclusion criteria were erythrodermic, pustular, palmoplantar, or other forms of psoriasis, psoriatic arthritis, concomitant onychomycosis proven by the KOH test and culture, skin conditions, and treatments at baseline that would interfere with psoriasis evaluation, immunosuppression, malignancies, autoimmune/genetic/metabolic/rheumatic diseases, and bacterial, viral, or fungal infection up to 4 weeks prior to inclusion in the study.

The diagnosis of psoriasis was confirmed in all cases by 2 dermatologists based on established clinical criteria [14]. Patients' age, gender, age at onset of psoriasis, skin psoriasis duration, family history of psoriasis, Psoriasis Area Severity Index (PASI) scores (0–72) [15], target Nail Psoriasis Severity Index (NAPSI) scores (0–32), and serum cytokine levels of TNF-*α*, IL-12/23 p40, and IL-17 were recorded. The evaluation of target NAPSI scores was performed as defined by Rich and Scher [16]. In the assessment of the target nail NAPSI scores, the fingernail representing the worst nail psoriasis was chosen. Ethics Board approval and written informed consent from all patients were provided.

Venous blood samples (5–10 mL) of all patients were collected in vacutainer tubes, without anticoagulant under sterile conditions between 09:00 and 11:30 am. After samples were rapidly centrifuged, serum was obtained and immediately stored at −70°C until batch processed. Serum cytokine levels of TNF-*α*, IL-12/23 p40, and IL-17 were determined by solid phase sandwich enzyme linked immunosorbent assay (R&D Systems Europe, Ltd). All assays were conducted according to manufacturer's protocols.

The objectives of this study were (1) to assess the serum levels of TNF-*α*, IL-12/23 p40, and IL-17 in psoriatic patients with and without nail involvement, (2) to detect possible statistically significant differences in the serum levels of TNF-*α*, IL-12/23 p40, and IL-17 between the psoriatic patients with and without nail involvement, and (3) to evaluate the correlations of the serum levels of TNF-*α*, IL-12/23 p40, and IL-17 with the target NAPSI score.

### 2.1. Statistical Analysis

Statistical analysis of the data was performed using the Statistical Package for Social Sciences (SPSS), version 22.0 (SPSS, Inc., Chicago, IL). Descriptive statistics were used to describe the study population's characteristics. All continuous variables were expressed as the mean ± standard deviation, median and range, while frequency distributions and percentages were used for categorical data. Fisher's exact test was used to compare dichotomous variables between psoriatic patients with and without nail involvement, while *t*-test or Mann-Whitney *U* test were used to compare continuous variables. Spearman's rank test was also used to explore relationships between continuous variables. Preliminary analyses were performed to ensure no violation of the assumptions of normality, linearity, and homoscedasticity. All tests were two sided, and the significance level was chosen to be *α* = 0.05.

## 3. Results and Discussion

In total, 52 patients with chronic plaque psoriasis were included in the study. Patients were studied and analyzed after they had been divided into 2 groups regarding the presence (*n* = 24) or not (*n* = 28) of nail psoriasis. Patients' demographic and clinical characteristics, as well as the serum levels of TNF-*α*, IL-12/23 p40, and IL-17 in each group, are summarized in Table 1.

The mean serum levels of TNF-*α* were significantly higher in the group of psoriatic patients presenting both cutaneous and nail lesions compared to those with only cutaneous lesions (*t*-test; 5.40 ± 1.17 versus 3.80 ± 1.63, *P* = 0.026). However, the median serum levels of IL-12/23 p40 in the group of psoriatic patients with both cutaneous and nail lesions did not significantly differ from those with only cutaneous lesions (Mann-Whitney *U* test; 92.52 (34.35–126.87) versus 150.68 (35.18–185.86), *P* = 0.297). Accordingly, the median serum levels of IL-17 presented no significant difference between the 2 groups (Mann-Whitney *U* test; 28.49 (0.00–28.49) versus 8.59 (0.00–8.59), *P* = 0.714).

The target NAPSI score presented high, positive statistically significant correlation with the serum levels of TNF-*α* (Spearman's rank test; Rho = 0.535, *P* = 0.01). On the contrary, target NAPSI score was not significantly correlated with either the serum levels of IL-12/23 p40 (Spearman's rank test; Rho = −0.346, *P* = 0.114) or the serum levels of IL-17 (Spearman's rank test; Rho = 0.056, *P* = 0.806).

Psoriasis is a common immune-mediated inflammatory disease that affects the skin, joints, and nails [17]. The pathogenesis of psoriasis is a complex interaction among genetic, immunological, and environmental components. There is an interplay between the innate and adaptive arms of the immune system in response to an unidentified trigger [17]. TNF-*α*, IL-12/23 p40, and IL-17 are critical checkpoints of inflammation in psoriasis [17]. Therefore, treatment of psoriasis has been revolutionized by targeting these inflammatory cytokines as key drivers of disease pathogenesis.

Recently, the view on the pathogenesis of psoriatic joint and nail disease has been challenged and a question has been raised of whether psoriatic skin disease shares the same pathogenetic principals as psoriatic joint and nail disease. The alternative pathogenetic model suggests that psoriatic nail and joint disease may be linked to tissue-specific factors that lead to activation of innate immune responses (autoinflammatory basis) rather than adaptive immunity (autoimmune basis) [18–21]. Additionally, van der Velden et al. cast doubt on whether nail psoriasis is a comorbidity of psoriasis or an isolated disease expression [22]. However, whatever the exact pathogenesis of nail psoriasis is, the disease seems to respond satisfactorily to biological agents [3, 7–13].

In literature, several studies have been conducted to evaluate the levels of various circulating cytokines in the serum of psoriatic patients and compared the results with those in healthy controls [23–32]. However, to our knowledge, no study has ever been conducted to assess and compare the serum levels of cytokines in psoriatic patients with and without nail involvement. Most studies have reported that the serum levels of TNF-*α* are significantly increased in patients with psoriasis compared with those of healthy controls [23–25, 28–30, 32]. Only Tigalonova et al. [31] and Jacob et al. [27] have found that the serum levels of TNF-*α* do not significantly differ between psoriatic patients and controls. The levels of IL-12 have been found to be significantly elevated in the serum of psoriatic patients compared with those of control [25, 26, 29], with the exception of Jacob et al. who reported decreased levels of IL-12 in the sera of psoriatic patients when compared with controls [27]. Last, Arican et al. presented no significant difference in the serum levels of IL-17 between psoriasis and control group [25].

In our study, 52 patients with chronic plaque psoriasis and PASI < 12 were included. Patients were studied and analyzed after they had been divided into 2 groups regarding the presence (*n* = 24) or not (*n* = 28) of nail psoriasis. Our intention was to design the study in a way that any elevation of the serum cytokine levels could be exclusively attributed to nail psoriasis, rather than to joint or extended skin psoriatic disease. After consideration, patients with psoriatic arthritis or moderate to severe chronic plaque psoriasis (PASI ≥ 12) were excluded, since serum levels of TNF-*α*, IL-12/23 p40, and IL-17 may potentially be elevated due to psoriatic skin, joint, or nail disease. Interestingly, the mean serum levels of TNF-*α* were significantly higher in the group of psoriatic patients presenting both cutaneous and nail lesions compared to those with only cutaneous lesions. On the other hand, neither the median serum levels of IL-12/23 p40 nor those of IL-17 presented any significant difference between the 2 groups. These results confirm the important role of TNF-*α* in the pathogenesis of nail psoriasis and may suggest that anti-TNF agents could be more beneficial on psoriatic nail disease than agents targeting IL-12/23 p40 or IL-17 and its receptors.

## 4. Conclusions

Nail involvement is a common manifestation of psoriasis and has started playing a major role in the overall assessment and management of psoriatic disease. The immunologically targeted approaches have progressively taken the place of most conventional therapies. Pharmaceutical companies have a variety of new biological agents for psoriatic disease in the pipeline for the next years. Our study showed that the mean serum levels of TNF-*α* were significantly higher in the group of psoriatic patients with nail lesions compared to those with only cutaneous lesions, contrary to the median serum levels of both IL-12/23 p40 and IL-17 that presented no significant difference between the 2 groups. Arising data on the role of various cytokines in psoriasis with or without nail involvement may influence the therapeutic decision among the different biologicals.

## Figures and Tables

**Table 1:** Patients' demographic and clinical characteristics, as well as the serum levels of TNF-*α*, IL-12/23 p40, and IL-17.

Characteristics	Psoriatic patients in total (*n* = 52)	Psoriatic patients with cutaneous and nail lesions (*n* = 24)	Psoriatic patients with only cutaneous lesions (*n* = 28)	*P* value^#^
Gender				
Male *n* (%)	22 (57.70)	12 (50.00)	10 (35.72)	0.662
Female *n* (%)	30 (42.30)	12 (50.00)	18 (64.28)	
Age (years)				
Mean ± SD	43.72 ± 12.44	48.12 ± 9.46	41.21 ± 13.53	0.218
Median (min–max)	44.50 (20.00–62.00)	29.00 (30.00–59.00)	42.00 (20.00–62.00)	
Disease duration (years)				
Mean ± SD	13.36 ± 9.93	20.12 ± 11.08	9.50 ± 6.99	0.012^*^
Median (min–max)	11.50 (1.00–40.00)	30.00 (10.00–40.00)	21.00 (1.00–22.00)	
Age at psoriasis onset (years)				
Mean ± SD	30.81 ± 14.11	29.25 ± 14.26	31.71 ± 14.48	0.704
Median (min–max)	32.00 (10.00–52.00)	39.00 (10.00–49.00)	42.00 (10.00–52.00)	
Family history				
Yes *n* (%)	21 (40.40)	9 (37.50)	12 (42.85)	0.889
No *n* (%)	31 (59.60)	15 (62.50)	16 (57.15)	
PASI score (0–72)				
Mean ± SD	4.63 ± 1.93	5.03 ± 2.15	4.40 ± 1.83	0.475
Median (min–max)	4.40 (2.10–9.00)	6.90 (2.10–9.00)	5.70 (2.20–7.90)	
Target NAPSI score (0–8)				
Mean ± SD	1.90 ± 2.84	3.70 ± 1.75	0.00	N/A
Median (min–max)	0.00 (0.00–8.00)	4.00 (2.00–8.00)	0.00	
TNF-*α* (pg/mL)				
Mean ± SD	4.38 ± 1.65	5.40 ± 1.17	3.80 ± 1.63	0.026^*^
Median (min–max)	4.16 (1.80–7.29)	3.13 (4.16–7.29)	5.33 (1.80–7.13)	
IL-12/23 p40 (pg/mL)				
Mean ± SD	79.33 ± 42.88	67.13 ± 32.77	86.30 ± 47.41	0.297
Median (min–max)	59.19 (34.35–185.86)	92.52 (34.35–126.87)	150.68 (35.18–185.86)	
IL-17 (pg/mL)				
Mean ± SD	4.27 ± 6.21	6.27 ± 9.49	3.13 ± 3.15	0.714
Median (min–max)	2.71 (0.00–28.49)	28.49 (0.00–28.49)	8.59 (0.00–8.59)	

^*^Statistically significant; ^#^comparing the variables between psoriatic patients with and without nail involvement.

## References

[1] Kyriakou A., Patsatsi A., Sotiriadis D. (2011). Detailed analysis of specific nail psoriasis features and their correlations with clinical parameters: a cross-sectional study. *Dermatology*.

[2] de Jong E. M., Seegers B. A., Gulinck M. K., Boezeman J. B. M., van de Kerkhof P. C. (1996). Psoriasis of the nails associated with disability in a large number of patients: results of a recent interview with 1728 patients. *Dermatology*.

[3] Langley R. G., Daudén E. (2010). Treatment and management of psoriasis with nail involvement: a focus on biologic therapy. *Dermatology*.

[4] Augustin M., Reich K., Blome C., Schäfer I., Laass A., Radtke M. A. (2010). Nail psoriasis in Germany: epidemiology and burden of disease. *British Journal of Dermatology*.

[5] Radtke M. A., Langenbruch A. K., Schafer I., Herberger K., Reich K., Augustin M. (2011). Nail psoriasis as a severity indicator: results from the PsoReal study. *Patient Related Outcome Measures*.

[6] Klaassen K. M. G., van de Kerkhof P. C. M., Pasch M. C. (2014). Nail Psoriasis, the unknown burden of disease. *Journal of the European Academy of Dermatology and Venereology*.

[7] Kyriakou A., Patsatsi A., Sotiriadis D. (2013). Biologic agents in nail psoriasis: efficacy data and considerations. *Expert Opinion on Biological Therapy*.

[8] de Vries A. C., Bogaards N. A., Hooft L. (2013). Interventions for nail psoriasis. *Cochrane Database of Systematic Reviews*.

[9] Langley R. G., Saurat J. H., Reich K. (2012). Recommendations for the treatment of nail psoriasis in patients with moderate to severe psoriasis: a dermatology expert group consensus. *Journal of the European Academy of Dermatology and Venereology*.

[10] Noiles K., Vender R. (2009). Nail psoriasis and biologics. *Journal of Cutaneous Medicine and Surgery*.

[11] Reich K., Burden A. D., Eaton J. N., Hawkins N. S. (2012). Efficacy of biologics in the treatment of moderate to severe psoriasis: a network meta-analysis of randomized controlled trials. *British Journal of Dermatology*.

[12] Reich K. (2009). Approach to managing patients with nail psoriasis. *Journal of the European Academy of Dermatology and Venereology*.

[13] Tan E. S. T., Chong W.-S., Tey H. L. (2012). Nail psoriasis: a review. *American Journal of Clinical Dermatology*.

[14] Mallbris L., Larsson P., Bergqvist S., Vingård E., Granath F., Ståhle M. (2005). Psoriasis phenotype at disease onset: clinical characterization of 400 adult cases. *The Journal of Investigative Dermatology*.

[15] Schmitt J., Wozel G. (2005). The psoriasis area and severity index is the adequate criterion to define severity in chronic plaque-type psoriasis. *Dermatology*.

[16] Rich P., Scher R. K. (2003). Nail psoriasis severity index: a useful tool for evaluation of nail psoriasis. *Journal of the American Academy of Dermatology*.

[17] Perera G. K., Di Meglio P., Nestle F. O. (2012). Psoriasis. *Annual Review of Pathology: Mechanisms of Disease*.

[18] McGonagle D. (2009). Enthesitis: an autoinflammatory lesion linking nail and joint involvement in psoriatic disease. *Journal of the European Academy of Dermatology and Venereology*.

[19] McGonagle D., Benjamin M., Tan A. L. (2009). The pathogenesis of psoriatic arthritis and associated nail disease: not autoimmune after all?. *Current Opinion in Rheumatology*.

[20] McGonagle D., Palmou Fontana N., Tan A. L., Benjamin M. (2010). Nailing down the genetic and immunological basis for psoriatic disease. *Dermatology*.

[21] McGonagle D., Tan A. L., Benjamin M. (2009). The nail as a musculoskeletal appendage—implications for an improved understanding of the link between psoriasis and arthritis. *Dermatology*.

[22] van der Velden H. M. J., Klaassen K. M. G., van de Kerkhof P. C. M., Pasch M. C. (2013). Fingernail psoriasis reconsidered: a case-control study. *Journal of the American Academy of Dermatology*.

[23] Abanmi A., Al Harthi F., Al Agla R., Khan H. A., Tariq M. (2005). Serum levels of proinflammatory cytokines in psoriasis patients from Saudi Arabia. *International Journal of Dermatology*.

[24] Abdel-Hamid M. F., Aly D. G., Saad N. E., Emam H. M., Ayoub D. F. (2011). Serum levels of interleukin-8, tumor necrosis factor-*α* and *γ*-interferon in Egyptian psoriatic patients and correlation with disease severity. *Journal of Dermatology*.

[25] Arican O., Aral M., Sasmaz S., Ciragil P. (2005). Serum levels of TNF-*α*, IFN-*β*, IL-6, IL-8, IL-12, IL-17, and IL-18 in patients with active psoriasis and correlation with disease severity. *Mediators of Inflammation*.

[26] Borska L., Andrys C., Krejsek J. (2008). Serum levels of the pro-inflammatory cytokine interleukin-12 and the anti-inflammatory cytokine interleukin-10 in patients with psoriasis treated by the Goeckerman regimen. *International Journal of Dermatology*.

[27] Jacob S. E., Nassiri M., Kerdel F. A., Vincek V. (2003). Simultaneous measurement of multiple Th1 and Th2 serum cytokines in psoriasis and correlation with disease severity. *Mediators of Inflammation*.

[28] Mussi A., Bonifati C., Carducci M. (1997). Serum TNF-alpha levels correlate with disease severity and are reduced by effective therapy in plaque-type psoriasis. *Journal of Biological Regulators and Homeostatic Agents*.

[29] Roussaki-Schulze A. V., Kouskoukis C., Petinaki E. (2005). Evaluation of cytokine serum levels in patients with plaque-type psoriasis. *International Journal of Clinical Pharmacology Research*.

[30] Takahashi H., Tsuji H., Hashimoto Y., Ishida-Yamamoto A., Iizuka H. (2010). Serum cytokines and growth factor levels in Japanese patients with psoriasis. *Clinical and Experimental Dermatology*.

[31] Tigalonova M., Bjerke J. R., Gallati H. (1994). Serum levels of interferons and TNF-*α* are not correlated to psoriasis activity and therapy. *Acta Dermato-Venereologica, Supplement*.

[32] Verghese B., Bhatnagar S., Tanwar R., Bhattacharjee J. (2011). Serum cytokine profile in psoriasis—a case-control study in a tertiary care hospital from Northern India. *Indian Journal of Clinical Biochemistry*.

